# Rare skin adverse reactions induced by osimertinib: a case report and literature review

**DOI:** 10.3389/fonc.2025.1523541

**Published:** 2025-01-23

**Authors:** Ye Zhang, Mingzhu Ling, Min Wang, Ye Chen, Liting Zhang

**Affiliations:** ^1^ Department of Pharmacy, The Second Affiliated Hospital of Zhejiang Chinese Medical University, Hangzhou, Zhejiang, China; ^2^ Department of Pulmonary and Critical Care Medicine, The Second Affiliated Hospital of Zhejiang Chinese Medical University, Hangzhou, Zhejiang, China

**Keywords:** adverse drug reaction, cutaneous vasculitis, osimertinib, EGFR-TKIs, case report

## Abstract

Osimertinib is a third-generation epidermal growth factor receptor tyrosine kinase inhibitor (EGFR-TKI) used in the treatment of EGFR mutation-positive advanced non-small cell lung cancer. Osimertinib-induced cutaneous vasculitis is a rare skin adverse reaction. We present a case study of a 49-year-old female who developed palpable purpura on her lower extremities on the 11th day of osimertinib treatment. Systemic involvement was not observed in the test results. The multidisciplinary team considered the clinical presentation of purpura as a potential case of cutaneous vasculitis. Osimertinib was immediately discontinued, and intravenous methylprednisolone along with oral cetirizine treatment was initiated. After 8 days since discontinuation of osimertinib, the patient’s skin purpura completely subsided. Subsequently, she was switched to almonertinib for treatment. We also conducted a literature review cutaneous vasculitis induced by osimertinib and other EGFR-TKIs. We hope to provide some safety alert information for clinical practice and recommend enhanced monitoring during the medication process.

## Introduction

Lung cancer is the leading cause of cancer-related deaths worldwide, with approximately 85% of cases attributed to non-small cell lung cancer (NSCLC) (1). Epidermal growth factor receptor (EGFR) belongs to a group of transmembrane receptors known as the HER/erbB family of receptor tyrosine kinases (RTK), which have the capability to homodimerize and/or heterodimerize with ligands, consequently activating the tyrosine kinase (TK) through autophosphorylation. This activation triggers downstream signaling pathways that ultimately promote tumor cell proliferation, angiogenesis, and migration (2, 3). Research suggested that more than 60% of NSCLC cases display elevated expression of EGFR, positioning EGFR as a promising target for NSCLC treatment (2). In 2004, research revealed a strong association between the efficacy of epidermal growth factor receptor tyrosine kinase inhibitor (EGFR-TKI) and EGFR gene mutations (4). Approximately 20% of NSCLC cases harbor EGFR mutations, with exon 19 deletions or exon 21 L858R mutations accounting for over 90% of these mutations (5). First-generation EGFR-TKIs such as gefitinib and erlotinib, as well as second-generation EGFR-TKIs like afatinib and dacomitinib, have shown significant clinical efficacy. However, the majority of patients develop resistance, with the most common mechanism being the T790M mutation (6). Osimertinib is a third-generation EGFR-TKI, approved by the U.S. Food and Drug Administration (FDA) in 2015 and launched in China in 2017 (7). Osimertinib has the capability to form irreversible covalent bonds with the cysteine residue at position 797 (C797) within the ATP-binding site of specific EGFR mutants, including T790M, L858R, and exon 19 deletions, effectively overcoming acquired resistance to first-generation and second-generation EGFR-TKIs (8). Skin toxicity is frequently observed since osimertinib also targets EGFR located in the skin epithelium (9). Retrospective data from the FDA Adverse Event Reporting System (FAERS) (10) indicate that the preferred terms for skin and subcutaneous tissue disorders caused by osimertinib mainly include nail disorder, onychoclasis, skin disorder, dermatitis acneiform, erythema multiforme, onychalgia, ingrowing nail, nail discoloration, and nail bed disorder. Here, we report a case of osimertinib-induced cutaneous vasculitis and review the literature on cutaneous vasculitis induced by osimertinib and other EGFR-TKI drugs, aiming to provide a reference for the safe use of osimertinib. Informed consent was obtained from the patient in advance.

## Case presentation

A 49-year-old Chinese female was diagnosed with lung adenocarcinoma in June 2021. She has no notable medical history, family history, or history of psychosocial disorders, and there is no relevant genetic information available. She subsequently underwent curative surgery followed by 2 cycles adjuvant chemotherapy, which she did not complete due to intolerance of chemotherapy-related adverse effects. In May 2024, the patient developed pleural effusion, and adenocarcinoma cells were identified in the pleural fluid, with immunohistochemistry suggesting pulmonary origin. Both the surgical specimen and pleural fluid underwent next-generation sequencing, which revealed EGFR exon 21 L858R point mutation. On June 4, 2024, the patient began monotherapy with osimertinib at 80 mg once daily, and during this period, she did not take any other medications.

**Figure 1:**
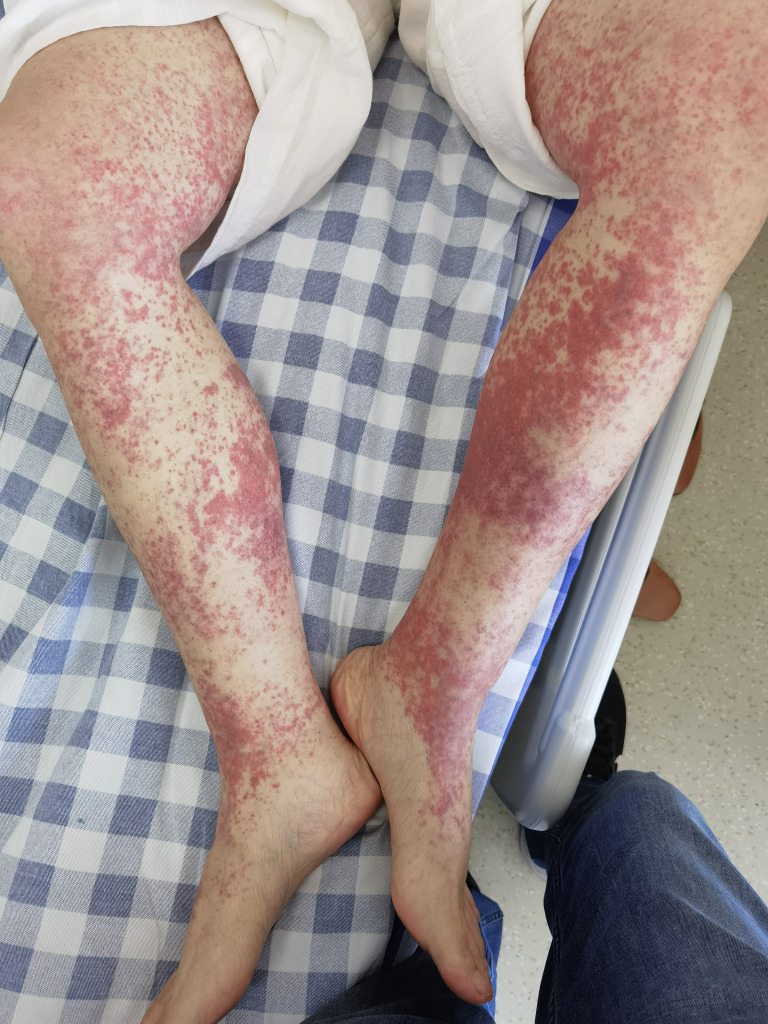
Palpable purpura, non-blanching over bilateral lower extremities.

Six days after starting osimertinib treatment, the patient developed multiple patchy red rashes, initially concentrated on the anterior chest and then gradually extending to the abdomen and lower extremities. The skin was slightly warm to touch with mild itching, which she could tolerate, and she continued the osimertinib treatment. By the eleventh day of osimertinib treatment, the rash on her chest and abdomen had subsided, but the palpable purpura on her lower extremities worsened. The non-blanching palpable purpura, slightly presenting as papules, covered more than 10% of the body surface area, without significant itching or pain, and no pustules (**Figure 1**). Additionally, the patient did not experience fever, joint pain or swelling, abdominal cramps, hematuria, or other symptoms indicative of connective tissue disease.

Laboratory tests showed that platelet count, serum immunoglobulin A, coagulation function, renal function indices (serum creatinine, urea, electrolytes), liver function indices (serum transaminases, bilirubin), and urinary red blood cells, anti-streptolysin O test, anti-cyclic citrullinated peptide antibodies were all within the normal range. Urine protein, proteinase-3 anti-neutrophil cytoplasmic antibody (ANCA), and myeloperoxidase-ANCA were all negative (**Table 1**). Unfortunately, the patient refused a skin biopsy.

**Table 1:** The patient’s laboratory test results.

Laboratory test	Results	Normal range
Platelet, ×10^9^/L	341	125-350
Serum Immunoglobulin A, g/L	2.6	0.7-4.0
Prothrombin time, s	13.1	9.2-13.9
Activated partial thromboplastin time, s	30.9	21.2-34.8
Urea, mmol/L	5.81	2.60-8.80
Creatinine, μmol/L	42.5	41.0-81.0
Serum potassium (K^+^), mmol/L	4.35	3.50-5.30
Serum sodium (Na^+^), mmol/L	137.6	137.0-147.0
Glutamic-pyruvic transaminase, U/L	14	7-40
Glutamic-oxalacetic transaminase, U/L	20	13-35
Total bilirubin, μmol/L	11.6	2.2-25.0
Urinary red blood cells,/μl	3	0-6
Urinary protein, mg/dl	Negative	Negative
Anti-streptolysin O test, IU/ml	43.5	0-116
Anti-cyclic citrullinated peptide antibodies, RU/ml	<0.5	0.0-5.0
Proteinase-3 anti-neutrophil cytoplasmic antibody (ANCA)	Negative	Negative
Myeloperoxidase-ANCA	Negative	Negative

Negative: the target substance was not detected, or the test result was within the normal reference range

Based on the clinical presentation of purpura, a multidisciplinary team consisting of dermatology, hematology, oncology, and pulmonology collectively diagnosed the condition as cutaneous vasculitis. Subsequently, the patient discontinued osimertinib and was treated with intravenous methylprednisolone at 40 mg/day, combined with oral cetirizine at 10 mg/day. Eight days after stopping osimertinib, the patient’s multiple skin purpura completely subsided, thus it was considered to be osimertinib-induced cutaneous vasculitis. However, considering the patient’s EGFR mutation and the significant adverse effects of post-operative adjuvant chemotherapy, with the patient’s consent, we opted to rechallenge with another EGFR-TKI.

**Figure 2:**
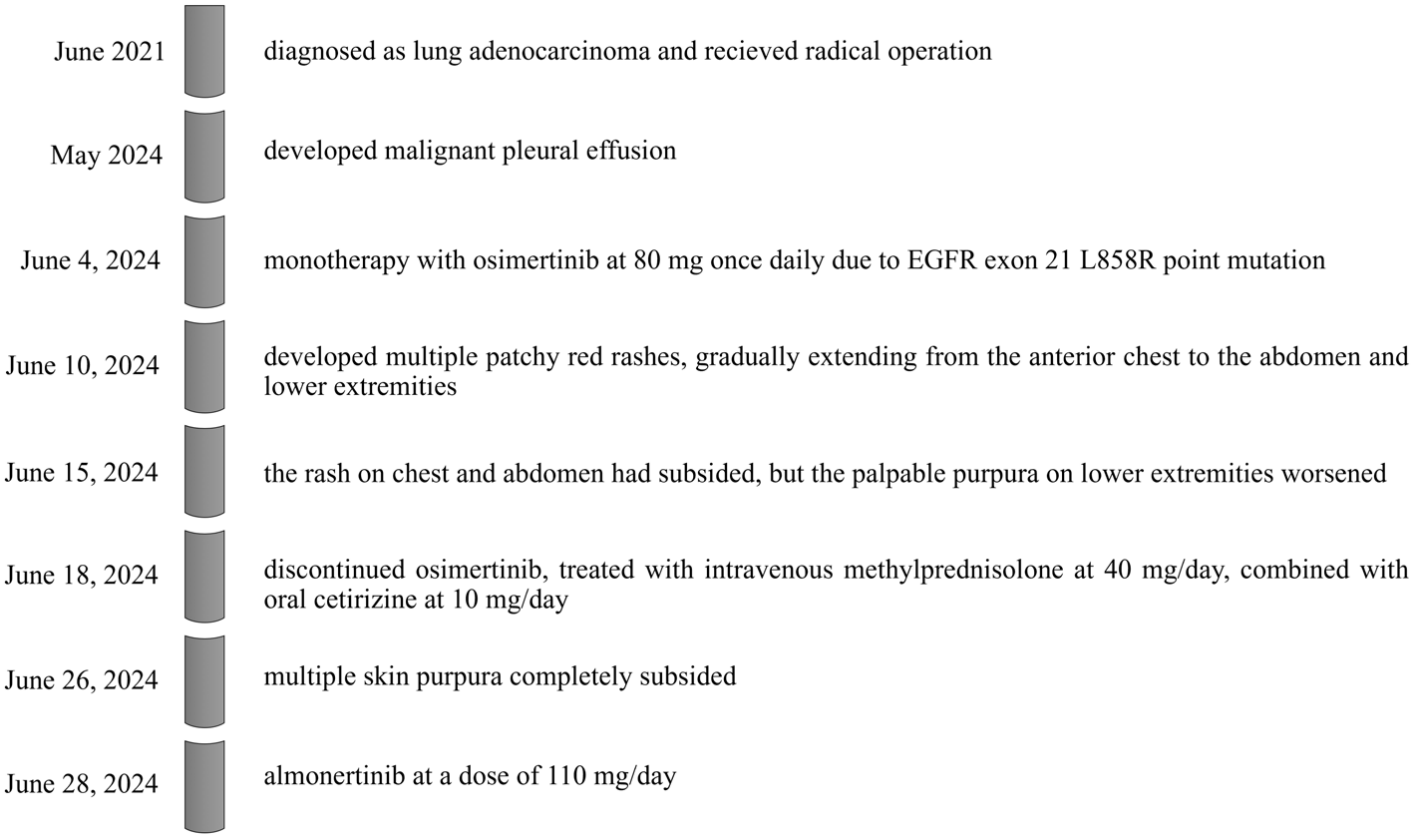
timeline of disease, intervention, and outcome.

We administered a third-generation EGFR-TKI, almonertinib, developed in China, at a dose of 110 mg/day. Six days after starting almonertinib, the patient developed a mild rash on the inner side of her forearm, which blanched upon pressure. After a few days of observation, the rash did not worsen and later resolved on its own. She continues to take almonertinib at 110 mg/day. The timeline is shown in **Figure 2**.

## Discussion

Cutaneous vasculitis is an inflammatory disease affecting the dermal blood vessel walls. According to the 2012 revised International Chapel Hill Consensus Conference Nomenclature of Vasculitides, it includes cutaneous components of systemic vasculitides, skin-limited variants of systemic vasculitides, and single-organ cutaneous vasculitis (11). It is estimated that approximately 15%-20% of cutaneous vasculitis is drug-induced, occurs most often within 7-21 days after initiation of the suspected drug (12).

The skin is most susceptible to small vessel vasculitis, with the typical manifestation being palpable purpura, and it can also present as petechiae, urticarial papules, vesicles, and pustules (11, 13). The subcategories of cutaneous small vessel vasculitis include Henoch-Schönlein Purpura, Acute hemorrhagic edema of infancy, urticarial vasculitis, erythema elevatum diutinum, and cryoglobulinemic vasculitis (14). Leukocytoclastic vasculitis is a common pathophysiological change caused by different etiologies, with skin changes often localized to the lower extremities (12, 15). To our knowledge, there have been four reported cases of osimertinib-induced cutaneous vasculitis (**Table 2**).

**Table 2:** Reported cases of osimertinib-induced cutaneous vasculitis.

Manuscript	Sex/Age	Time to onset after osimertinib treatment	Cutaneous vasculitis type	Whether to discontinue osimertinib	Treatment and outcome	Subsequent treatment
Hamada et al. (2019) (16)	Female/65	6 weeks	cutaneous vasculitis	Yes	prednisolone (25 mg/d, tapered to 7.5 mg/d), purpura disappeared	osimertinib rechallenge at same dose, no vasculitis recurrence
Calderon et al. (2022) (17)	Female/86	19 days	cutaneous vasculitis	Yes	corticosteroids (120 mg/d for 6 days and 60 mg/d) with cyclophosphamide (500 mg every 2 weeks for 3 injections), purpura regressed after 8 days, disappeared after 33days	unknown
Iriarte et al. (2022) (18)	Female/45	5 months	leukocytoclastic vasculitis	No	dapsone (50 mg/d for 2 weeks, tapered down over 6 weeks), purpura disappeared after 2 weeks	continued osimertinib
Weng et al. (2024) (19)	Male/50	1 week	urticarial vasculitis	Yes	methylprednisolone (0.4 mg/kg/d) for 7 days, skin lesions resolved after 1 month	osimertinib rechallenge, beginning with half dose with a gradual titration to 80 mg/d

Manuscript: the authors (publication year) and references. Cutaneous vasculitis type: skin biopsy results.

We searched the PubMed, MEDLINE, Elsevier ScienceDirect, and Web of Science databases to investigate and review existing literature reports and review articles on cutaneous vasculitis induced by EGFR-TKIs. Over the past two decades, besides osimertinib, the primary EGFR-TKI drugs causing cutaneous vasculitis have been the first generation EGFR-TKIs, gefitinib and erlotinib, with a total of 14 cases reported (**Table 3**).

**Table 3:** Reported cases of other EGFR-TKIs-induced cutaneous vasculitis.

Manuscript	EGFR-TKI	Sex/Age	Time to onset after treatment	Cutaneous vasculitis	Whether to discontinue EGFR-TKI	Treatment and outcome	Subsequent treatment
Kurokawa et al. (2005) (20)	gefitinib	Female/76	77 days	leukocytoclastic vasculitis	Yes	betamethasone (2 mg/d), subsided to brown pigmentation	no rechallenge
Fernandez-Guarino et al. (2007) (21)	gefitinib	Male/62	2 months	necrotizing vasculitis	Yes	no treatment, disappeared after 3 weeks	unknown
Uchimiya et al. (2010) (22)	gefitinib	Female/74	1 month	leukocytoclastic vasculitis	Yes	no treatment, disappeared after 2 weeks	gefitinib rechallenge intermittently, no vasculitis recurrence
Uchimiya et al. (2010) (22)	gefitinib	Female/76	2 months	leukocytoclastic vasculitis	Yes	no treatment, disappeared after 17 days	gefitinib rechallenge, no vasculitis recurrence
Nozato et al. (2010) (23)	gefitinib	Female/52	About 2 months	Henoch-Schönlein purpura	Yes	topical steroids, disappeared	gefitinib rechallenge, no vasculitis recurrence
Ko et al. (2011) (24)	gefitinib	Male/47	4 months	necrotizing vasculitis	Yes	prednisolone (20 mg/d), disappeared after 2 weeks	gefitinib rechallenge, vasculitis recurred
Boeck et al. (2007) (25)	erlotinib	Male/67	4 weeks	leukocytoclastic vasculitis	Yes	prednisolone (40mg for 3 days and 30-20-10mg from day 4 to 6) with topical anti-infective therapy, disappeared after 6 weeks	unknown
Boeck et al. (2007) (25)	erlotinib	Female/70	2 weeks	leukocytoclastic vasculitis	Yes	prednisolone (40 mg for 3 days and 30-20-10 mg from day 4 to 6) with topical therapy, disappeared after 5 weeks	unknown
Yuba et al. (2010) (26)	erlotinib	Female/68	3 months	Henoch-Schönlein purpura	No(reduced dose)	improved	continue erlotinib
Takahashi et al. (2011) (27)	erlotinib	Female/69	8 weeks	cutaneous vasculitis	Yes	no treatment, disappeared after 2 weeks	unknown
Su et al. (2012) (28)	erlotinib	Female/50	8 days	leukocytoclastic vasculitis	Yes	no treatment, disappeared after 7 weeks	erlotinib rechalleng(100 mg/d),no vasculitis recurrence
Brandi et al. (2013) (29)	erlotinib	Male/69	2 weeks	leukocytoclastic vasculitis	Yes	no treatment, disappeared after 2 weeks	erlotinib rechalleng(100 mg/d),no vasculitis recurrence
Sawada et al. (2016) (30)	erlotinib	Female/78	About 80 days	leukocytoclastic vasculitis	Yes	no treatment, disappeared after 2 weeks	erlotinib rechalleng(100 mg/d),no vasculitis recurrence
Fekete et al. (2019) (31)	erlotinib	Female/58	8 months	leukocytoclastic vasculitis	Yes	prednisolone (1 mg/kg for 2 weeks, reduce 5mg every 3 days) with topical steroid and antibiotic therapy (once daily), disappeared within 7 weeks	erlotinib rechalleng(100 mg/d),no vasculitis recurrence

Manuscript: the authors (publication year) and references. Cutaneous vasculitis type: skin biopsy results.

Currently, there are 18 reported cases of EGFR-TKI (Osimertinib, Gefitinib, Erlotinib) induced cutaneous vasculitis. Among these, there are 14 cases of lung cancer, 2 cases of advanced hepatocellular carcinoma, 1 case of metastatic pancreatic cancer, and 1 case of adenoid cystic carcinoma of the maxilla with multiple nodules in both lungs after surgery and radiotherapy. There are 13 female patients (72.2%) and 5 male patients (27.8%), with a median age of 65 ± 12 years, ranging from 45 to 86 years. The average time from the start of EGFR-TKI treatment to the onset of cutaneous vasculitis is 8.7 ± 7.8 weeks, with a range of 1 week to 8 months, indicating that clinical manifestations and signs may have a delayed onset. Almost all patients had clinical symptoms confined to the skin with no other systemic manifestations.

Drug-induced skin-limited vasculitis is typically characterized as cutaneous small vessel vasculitis, with a temporal association between the onset of symptoms and drug administration, and reversibility of symptoms upon suspected drug discontinuation (11). The pathogenesis of drug-induced cutaneous small vessel vasculitis is complex and generally considered to be a Type III hypersensitivity reaction. The drug acting as a hapten, binding to large molecules in the body to form immune complexes that deposit in small vessels, triggering complement cascade reactions, leading to the release of C3a and C5a, activation of inflammatory responses, neutrophil chemotaxis, and endothelial activation, ultimately leading to vascular damage (32–34). More than 50 drugs have been reported in the literature as potential inducers of cutaneous small vessel vasculitis, including antibiotics, NSAIDs, TNF-inhibitors, warfarin, immune checkpoint inhibitors, and others (14, 35). The mechanism by which EGFR-TKIs induced cutaneous vasculitis is not yet clear but is believed to be similar to other forms of cutaneous small vessel vasculitis, potentially mediated by immune complexes (21). In addition to being found in tumor cells, EGFR is also expressed in the endothelial cells of skin vessels. This has led some researchers to speculate that the skin toxicity observed with EGFR-TKIs may not be immune reaction-related, but rather associated with the inhibition of EGFR itself. Inhibition of the EGFR signaling pathway can potentially trigger endothelial inflammation, disrupt vascular tone, and increase vascular permeability. These effects may ultimately manifest as skin purpura and induce vasculitis (18, 21, 36). Some researchers have suggested that it may exhibit a dose-dependent pattern (31). Research on skin purpura has found that the inhibition of endothelial cell proliferation by EGFR-TKI is time-dependent. To compensate for the EGFR pathway in endothelial cells, IQGAP1, which is involved in various signaling pathways including cell-cell adhesion, may increase. Overexpression of IQGAP1 may disrupt adhesion junctions, leading to red blood cell extravasation (37).

In general, the most crucial step in managing drug-induced vasculitis is discontinuing the suspected drug, which typically leads to spontaneous resolution within days to weeks (33). Since cutaneous small vessel vasculitis is typically confined to the skin, supportive care is the mainstay of treatment. This includes rest, leg elevation, compression stockings, local application of corticosteroids, and NSAIDs (13). For severe symptoms, systemic corticosteroids may be administered, with a recommended dosage of 0.5-1 mg/kg/day of prednisone or an equivalent corticosteroid (13, 14). Other first-line treatment options include colchicine, dapsone, and azathioprine (13, 38, 39). Some researchers suggested that dapsone is an effective targeted therapy for cutaneous leukocytoclastic vasculitis that does not compromise the immune system and allows the continuation of EGFR-TKI therapy (18). Additionally, if urticarial symptoms are present, antihistamines can be used to alleviate itching.

In cases where patients have experienced drug hypersensitivity reactions, subsequent treatment options typically involve either using a non-cross-reacting alternative or undergoing desensitization therapy with the allergenic drug. Desensitization therapy involves gradually increasing the dosage of the drug, which can help patients develop temporary tolerance to the medication. This approach allows them to safely undergo drug treatment without triggering the hypersensitivity reaction (32). Among the 18 cases of EGFR-TKI-induced cutaneous vasculitis listed in the previous tables, 16 cases (88.9%) discontinued the EGFR-TKI. One case did not discontinue osimertinib and achieved complete remission with dapsone treatment without recurrence. Another case improved and did not recur after a dose reduction of erlotinib. Among the 16 cases discontinued TKI, after treatment, 3 cases were rechallenged with the original dose, and 6 cases rechallenged with a reduced dose or intermittent use of the original drug, all without recurrence of cutaneous vasculitis. However, there was 1 case experienced a recurrence of cutaneous vasculitis after rechallenge. One case did not rechallenge the original or similar drugs, and there was no mention of subsequent antitumor therapy in 5 cases.

Almonertinib is a structural modification of osimertinib, with the difference being that the indole 1-position substituent in almonertinib is a cyclopropyl group, while in osimertinib it is a methyl group (40). There has been a report in the literature of a patient who experienced severe rash with osimertinib was successfully switched to almonertinib (41). In our case, due to patient compliance, osimertinib was not rechallenged. After switching to the same class of EGFR-TKI, namely almonertinib, the patient experienced a mild, self-limiting rash and did not exhibit any further clinical signs of cutaneous vasculitis. This allowed for the continued use of almonertinib without any adverse reactions. Based on these observations, we lean towards considering cutaneous vasculitis caused by EGFR-TKIs is a drug-induced hypersensitivity reaction. The fact that altering the medication has allowed for the safe continuation of treatment further supports this conclusion.

A previous meta-analyses on gefitinib and erlotinib indicated that EGFR-TKI-associated rash may serve as a clinical marker for predicting the effective treatment response to EGFR-TKIs in NSCLC patients, including objective response rate (ORR) and disease control rate (DCR). Additionally, patients who develop a rash tend to have longer progression-free survival (PFS) and overall survival (OS) (42). The rash induced by EGFR-TKIs could be an external manifestation of their therapeutic efficacy. Similar results were also found in the research of the second-generation EGFR-TKIs afatinib and dacomitinib (43, 44). Nevertheless, it remains unclear whether skin adverse events induced by third-generation EGFR-TKIs, such as osimertinib, can serve as predictive indicators of their antitumor efficacy.

Our study has certain limitations that need to be acknowledged. First, we were unable to conduct a pathological examination to confirm the presence of cutaneous vasculitis due to the patient’s refusal for a skin biopsy. Instead, the case was managed by a multidisciplinary team based on clinical presentation and other available diagnostic information. It is important to note that skin lesions play a crucial role in the diagnosis of vasculitis, and the absence of a biopsy may impact the accuracy and certainty of the diagnosis (12). The presence of palpable purpura on the patient’s lower extremities is in line with the characteristic features of cutaneous small vessel vasculitis. These features include the extravasation of red blood cells, resulting in non-blanching purpura, the influence of gravity on the deposition of immune complexes that primarily affect the lower legs, and the indication of inflammatory cell infiltration (12, 13). In a study involving 32 patients with NSCLC who experienced purpuric drug eruptions due to EGFR-TKIs (gefitinib, erlotinib, and afatinib), the predominant characteristics of these eruptions were observed to be purpuric papules, pustules, or confluent plaques on the lower limbs. Pathological examination confirmed leukocytoclastic vasculitis in only 3 patients, while 63% (20/32) of the patients tested positive for *Staphylococcus aureus* in skin tests. All patients received systemic antimicrobial treatment (36). Furthermore, a case report documented a patient who developed a purpuric drug eruption attributed to afatinib. The patient exhibited purpura and pustules on the legs, accompanied by an ulcer and a crust. *Pseudomonas aeruginosa* and coagulase-negative *Staphylococcus* were detected in the pustules, while the skin pathology did not indicate vasculitis. The patient received systemic anti-infective treatment, which resulted in positive outcomes (45). In our case, the patient did not exhibit pustules, ulcers, or any comparable symptoms, nor did she receive any antimicrobial drugs during her treatment. Significantly, the purpura completely resolved. As a result, the possibility of a purpuric drug eruption is diminished, while the likelihood of cutaneous vasculitis as the diagnosis becomes more prominent. Moving forward, it is imperative to delve deeper into the mechanisms underlying cutaneous vasculitis, identify specific biomarkers to facilitate diagnosis, alleviate the discomfort associated with skin biopsies for patients, and enhance patient compliance.

## Conclusion

Our case can provide some safety alert information for clinical practice. Cutaneous vasculitis is a rare skin adverse reaction to EGFR-TKIs, and it is crucial to remain vigilant, identify it early, educate the patient, and manage it effectively. Additionally, skin adverse reactions may be related to the efficacy of EGFR-TKIs, but their predictive role warrants further investigation.

## Data Availability

The raw data supporting the conclusions of this article will be made available by the authors, without undue reservation.
